# Non-spike and spike-specific memory T cell responses after the third dose of inactivated COVID-19 vaccine

**DOI:** 10.3389/fimmu.2023.1139620

**Published:** 2023-04-11

**Authors:** Ruoqiong Huang, Liyang Ying, Jiangmei Wang, Jie Xia, Yanjun Zhang, Haiyan Mao, Ruoyang Zhang, Ruoxi Zang, Zhenkai Le, Qiang Shu, Jianguo Xu

**Affiliations:** ^1^ Children’s Hospital, Zhejiang University School of Medicine, National Clinical Research Center for Child Health, Hangzhou, Zhejiang, China; ^2^ Department of Microbiology, Zhejiang Provincial Center for Disease Control and Prevention, Hangzhou, Zhejiang, China

**Keywords:** COVID-19, inactivated vaccines, CoronaVac, T cell responses, spike, non-spike

## Abstract

**Background:**

During the COVID-19 epidemic, vaccination has become the most safe and effective way to prevent severe illness and death. Inactivated vaccines are the most widely used type of COVID-19 vaccines in the world. In contrast to spike-based mRNA/protein COVID-19 vaccines, inactivated vaccines generate antibodies and T cell responses against both spike and non-spike antigens. However, the knowledge of inactivated vaccines in inducing non-spike-specific T cell response is very limited.

**Methods:**

In this study, eighteen healthcare volunteers received a homogenous booster (third) dose of the CoronaVac vaccine at least 6 months after the second dose. CD4^+^ and CD8^+^ T cell responses against a peptide pool from wild-type (WT) non-spike proteins and spike peptide pools from WT, Delta, and Omicron SARS-CoV-2 were examined before and 1-2 weeks after the booster dose.

**Results:**

The booster dose elevated cytokine response in CD4^+^ and CD8^+^ T cells as well as expression of cytotoxic marker CD107a in CD8^+^ T cells in response to non-spike and spike antigens. The frequencies of cytokine-secreting non-spike-specific CD4^+^ and CD8^+^ T cells correlated well with those of spike-specific from WT, Delta, and Omicron. Activation-induced markers (AIM) assay also revealed that booster vaccination elicited non-spike-specific CD4^+^ and CD8^+^ T cell responses. In addition, booster vaccination produced similar spike-specific AIM^+^CD4^+^ and AIM^+^CD8^+^ T cell responses to WT, Delta, and Omicron, indicting strong cross-reactivity of functional cellular response between WT and variants. Furthermore, booster vaccination induced effector memory phenotypes of spike-specific and non-spike-specific CD4^+^ and CD8^+^ T cells.

**Conclusions:**

These data suggest that the booster dose of inactive vaccines broadens both non-spike-specific and spike-specific T cell responses against SARS-CoV-2.

## Introduction

Inactivated viral vaccines have been successfully developed for decades around the world in immunization programs such as polio and influenza. Vaccines of this type are created by inactivating the whole pathogen through physical or chemical processes. The processing stops the pathogen’s capacity to replicate in the vaccinated hosts, but the human immune system can still respond to the vaccines (1). As of 8 February 2023, the World Health Organization (WHO) has accepted three inactivated COVID-19 vaccines, including Covaxin (Bharat Biotech, India), Covilo (Sinopharm, China), CoronaVac (Sinovac, China), for emergency use. Covilo and CoronaVac vaccines have been approved by 93 and 56 countries, respectively (https://covid19.trackvaccines.org/agency/who/). They are also the most easily obtainable COVID-19 vaccines, particularly in developing countries. Covilo and CoronaVac vaccines account for more than half of the delivered doses in the world (2).

The SARS-CoV-2 Omicron variant (B.1.1.529), first reported in South Africa in November 2021 (3), has been the dominant variant globally. It has more than 30 mutations in the spike gene compared to the ancestral strain and is associated with elevated infectivity and immune evasion (4). There are also approximately 20 mutations in the conserved non-spike genome, which is less significant for infectivity and immune evasion (5). Fortunately, the Omicron variant results in lower hospitalization and death rates than the previous strains (6). Up to the present, the Omicron variant has many sub-lineages – BA.1, BA.1.1, BA.2, BA.3, BA.4, and BA.5, BA.4.6, BF.7, BQ.1, BQ.1.1, XBB.1, and XBB.1.5, which will continue to evolve in the near future. The SARS-CoV-2 Omicron variant can escape from humoral immunity due to the mutations in the receptor binding domain (RBD) of spike protein (S), which is essential for the binding for neutralization antibodies (7). However, escape from cell immunity of the Omicron variant has not been documented in the literature.

SARS-CoV-2 virus consists of four structural proteins including S, envelope (E), membrane (M) and nucleocapsid (N); and other non-structural proteins. It has been reported that strong CD4^+^ and CD8^+^ T cell memory responses against both spike and non-spike antigens were elicited in convalescent patients with SARS-CoV-2 infection (8). In COVID-19 convalescent patients, there was a significant correlation between the percentage of SARS-CoV-2 non-spike-specific CD4^+^ T cells and anti-spike RBD IgG. Non-spike peptides stimulated cytokine secretion in CD4^+^ T cells (9). Lang-Meli et al. demonstrated that CD8^+^ T cells targeted both spike and non-spike epitopes, with non-spike-specific epitopes being dominant, in COVID-19 convalescent individuals (10). Naranbhai et al. showed that spike-specific CD4^+^ T cell response was maintained against Omicron, while spike-specific CD8^+^ T cell response was decreased (11). Therefore, CD4^+^ and CD8^+^ T cell responses against both non-spike and spike antigens play an essential role in SARS-CoV-2 infection.

Most existing studies of inactivated COVID-19 vaccines focus on humoral and cellular immune responses to spike antigens. The effect of inactivated COVID-19 vaccines on non-spike-specific T cell response is poorly understood. In the present study, peripheral blood mononuclear cells (PBMCs) were collected from individuals before and after the homogenous booster (third) dose of CoronaVac COVID-19 vaccine. We compared non-spike and spike-specific cellular responses of circulating CD4^+^ and CD8^+^ T cells.

## Materials and methods

### Human study subjects

This is an observational vaccine study conducted during China national COVID-19 vaccination campaign. We enrolled 18 volunteers from a cohort of health care workers at Children’s Hospital of Zhejiang University School of Medicine. All of the volunteers enrolled in the study had body mass index in the normal range (18.5 to 24.9) and were in good physical status without significant co-morbidities. The volunteers had received two doses of SARS-CoV-2 inactivated vaccine CoronaVac between February 23, 2021 and May 28, 2021. Subjects were excluded from the study if they had a positive SAR-CoV-2 test at any time before the study period. The study protocol was approved (EC/IRB approval number: 2021029) by the ethics committee of the Children’s Hospital of Zhejiang University School of Medicine (Hangzhou, China) and conducted according to the provisions detailed in Declaration of Helsinki. Written informed consent was acquired from each volunteer. A third dose of CoronaVac was administered to volunteers more than 6 months following the second dose between November 16, 2021 and January 18, 2022. Whole blood samples were collected from the volunteers before the booster dose and 1-2 weeks after.

### Isolation of PBMCs

Peripheral blood samples were dispensed into heparin tubes and processed within 4 hours after collection. PBMCs were isolated from the whole blood *via* density gradient centrifugation using Lymphocyte Separation Medium (TBD, Tianjin, China) per the manufacturer’s instructions. PBMCs were counted *via* trypan blue staining, cryopreserved in pre-cooled fetal bovine serum (FBS, Biological Industries, Kibbutz Beit-Haemek, Israel) with 10% DMSO (Sigma-Aldrich, Burlington, MA), and stored in liquid nitrogen until experiment.

### Interferon-γ enzyme-linked immunospot (ELISpot) assays

ELISpot assay was performed using ELISpot Plus: Human IFN-γ (ALP) Kit (Mabtech, Sweden) according to the manufacturer’s protocol. Before the assay, PBMCs were thawed and rested overnight at 37°C humidified incubator with 5% CO_2_ in RPMI medium 1640 supplemented with 10% FBS. After preparation of ELISpot plates according to the protocol, 2×10^5^ PBMCs were added to each well in duplicate. Cells were stimulated with spike peptide pools (15 mers with 11 amino acid overlap) from wild-type (WT), Delta (B.1.617.2), and Omicron BA.1 (B.1.1.529) variant (GenScript, Piscataway, NJ) as well as a non-spike peptide pool containing N protein, M protein, and open reading frame proteins (ORF) of WT SARS-CoV-2 (Mabtech) at a final concentration of 2 µg/mL for 20 h at 37°C. No stimulation (DMSO only) and anti-CD3 stimulation were served as negative and positive control, respectively. Anti-CD28 (Mabtech) was added at a final concentration of 0.1 µg/mL to enhance antigen-specific responses. The plates were rinsed with Phosphate-buffered saline (PBS), incubated with Biotinylated mouse anti-human IFN-γ antibody (7-B6-1-biotin, 1 µg/mL) for 2 h, and rinsed with PBS again. The plates were then washed a second time and incubated for 1 h with Streptavidin-ALP (1:1000). After rinsing the plates, the cells were developed with 100 µL of BCIP/NBT-plus substrate solution until distinct spots were visible (usually 10-20 min). Color development was stopped by washing extensively in tap water. The plates were dried in a dim place for 2-3 days. ELISpot plates were counted *via* Mabtech ELISpot/FluoroSpot readers (Mabtech IRIS). The data were subtracted by the background value in the DMSO stimulation and were expressed as spot-forming unit (SFU)/10^6^ PBMCs.

### Flow cytometry-based assay for T cell response and cytokine-producing cells

PBMCs were thawed and rested overnight at 37°C humidified incubator with 5% CO_2_ in RPMI medium 1640 supplemented with 10% FBS. A total of 1 × 10^6^ PBMCs was stimulated with the spike peptide pools from WT, Delta (B.1.617.2), and Omicron BA.1 (B.1.1.529) as well as the non-spike peptide pool from wild-type SARS-CoV-2 for 20 h at a concentration of 2 µg/mL of final concentration. No stimulation (DMSO only) and anti-CD3 stimulation served as the negative and positive control, respectively. Anti-CD28 (BioLegend, San Diego, CA) and anti-CD49d (BioLegend) were added to all wells at a final concentration of 1 µg/mL to enhance antigen-specific responses and incubated for 20 h at 37°C. Subsequently, protein transport inhibitor GolgiPlug™ (containing Brefeldin A, BD Biosciences, NJ) and GolgiStop™ (containing Monensin, BD Biosciences) were added to cells at a dilution of 1:1000 and 1:1500, respectively. At the same time, BV421 anti-human CD107a (BioLegend) was added to cells. The incubation was continued for 4 h at 37°C.

For flow cytometry, cells were resuspended with cold flow cytometry buffer (PBS, 0.5% BSA) and stained with Fixable Viability Dye eFluor™ 780 (Thermo Fisher, Waltham, MA) for 30 min. After washing with flow cytometry buffer, cells were labelled with BV510 anti-human CD14 (BD Biosciences), FITC anti-human CD3 (Thermo Fisher), BV650 anti-human CD8 (BD Biosciences), AF700 anti-human CD4 (BioLegend), BV605 anti-human CD45RA (BioLegend), PerCP-cy5.5 anti-human CCR7 (BioLegend), PE anti-human CD137 (BioLegend), BV785 anti-human CD69 (BioLegend), and APC anti-human OX40 (BioLegend) or the isotype control at 4°C for 30 min. For intracellular staining, cells were incubated with Fixation/Perm working buffer (Thermo Fisher) for 30 min. The cells were then washed with flow cytometry buffer, resuspended in the perm diluent, and stained with BV711 anti-human IFN-γ (BioLegend**)** and PE-cy7 anti-human TNF-α (BioLegend) or the isotype control at 4°C for 30 min. After washing with perm diluent, PBMCs were resuspended with 300 µL flow cytometry buffer and examined *via* BD LSRFortessa™ flow cytometer. The data were analyzed using FlowJo V10 software.

### Data analysis and statistics

Data are shown as mean ± standard error of the mean (SEM). Statistical analysis was performed using the GraphPad Prism V8.0 software (GraphPad, San Diego, CA). Data were tested for normality using the Anderson-Darling test, the D′Agostino-Pearson Omnibus normality test, the Shapiro-Wilk normality test, and the Kolmogorov-Smirnov test with Dallal-Wilkinson-Lilliefor corrected p value. To compare two groups, Wilcoxon matched-pairs signed rank test was performed for nonparametric data. Comparisons of multiple groups were performed *via* RM one-way ANOVA with Holm-Šídák’s multiple comparisons test for parametric data or Friedman test with Dunn’s multiple comparisons test for nonparametric data.

## Results

### Increased IFN-γ ELISpot response against spike and non-spike antigens after a third (booster) dose of CoronaVac

**Figure 1:**
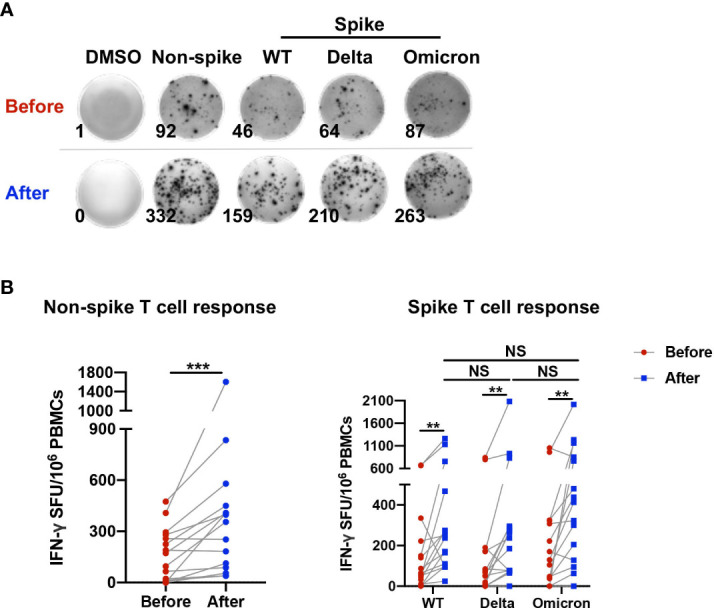
T cell reactivity to non-spike and spike antigens induced by a third dose of CoronaVac. PBMCs obtained before and 1-2 weeks after the third dose of CoronaVac were stimulated with spike peptide pools from WT, Delta, and Omicron as well as a non-spike peptide pool from WT SARS-CoV-2 for 20 h. ELISpot assay was performed on PBMCs. No stimulation (DMSO only) was served as negative control. **(A)** Representative photos of ELISpot wells were presented. **(B)** The number of IFN-γ-producing T cells was enumerated as the spot-forming units (SFU) per 10^6^ PBMCs. Points and connecting lines represent raw data for a single participant. Data were normalized against the background value in the DMSO stimulation. Comparisons were performed *via* Wilcoxon matched-pairs signed rank test between two groups or Friedman test with Dunn’s multiple comparisons test among multiple groups. n = 15. **p < 0.01, ***p < 0.001. NS, not significant.

The characteristics of study participants and are shown in **Table 1**. To determine the T cell reactivity to non-spike and spike antigens induced by a third dose of CoronaVac, the number of IFN-γ-secreting cells in PBMCs obtained before and 1-2 weeks after the booster dose was examined *via* ELISpot assay (**Figure 1A**). The booster dose significantly increased the frequency of IFN-γ producing cells upon stimulation with the non-spike peptide pool derived from N, M, and ORF proteins of wild-type SARS-CoV-2 (**Figure 1B**). Similarly, the number of IFN-γ-secreting cells in PBMCs was elevated after the third dose in response to spike peptide pools from WT, Delta, and Omicron. However, no significant difference was observed among WT, Delta, and Omicron (**Figure 1B**).

**Table 1:** Characteristics of donor cohort.

	Vaccinees (n = 18)
Age, y	23-47 (Median = 31, IQR = 9)
Gender, %	
Male	33 (6/18)
Female	67 (12/18)
Duration of post-vaccination (days)	9-14 (Median = 10.5, IQR = 3)

### Elevated function of non-spike and spike-specific CD4^+^ and CD8^+^ T cells after the third dose

**Figure 2:**
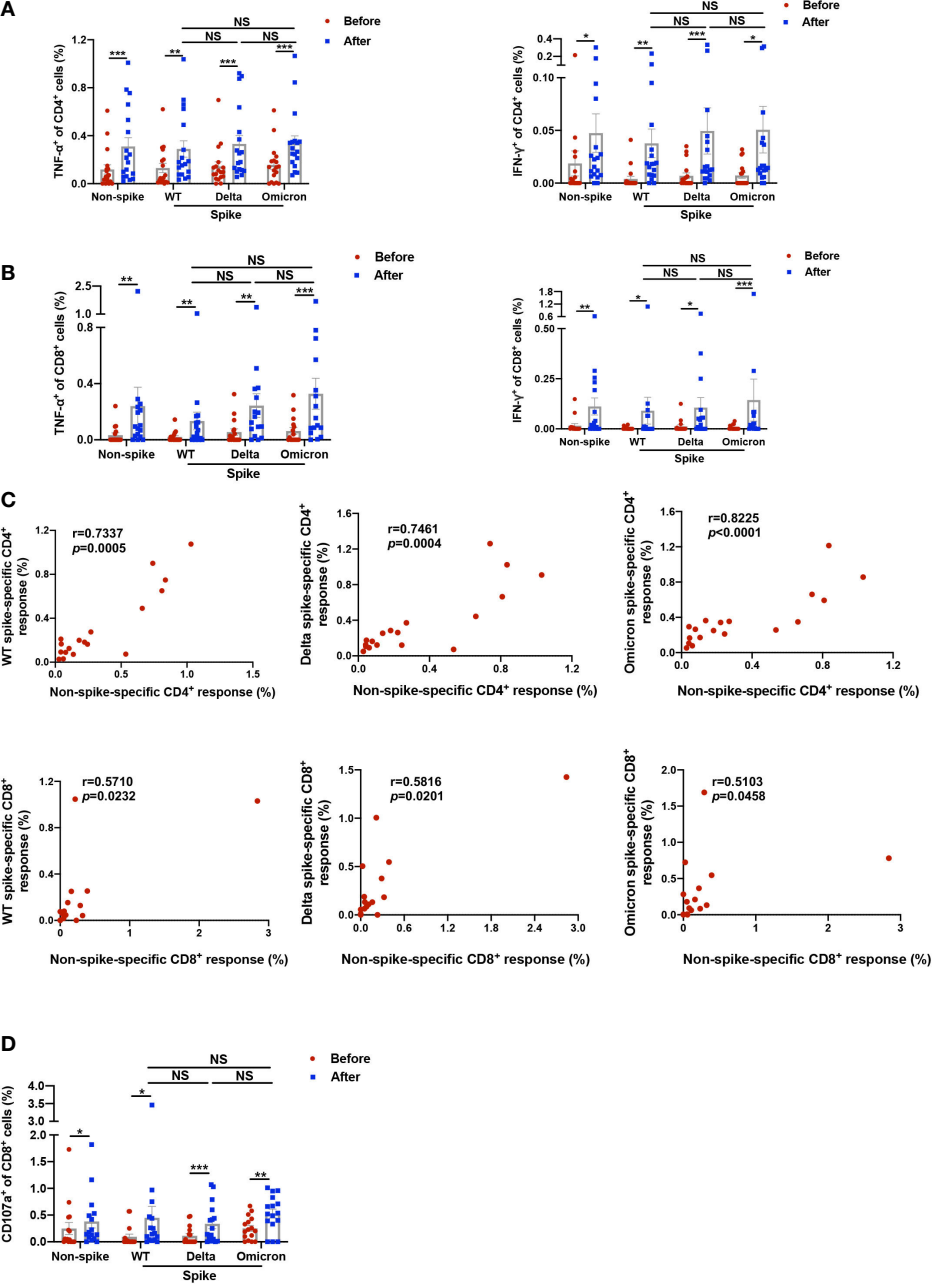
Functional analysis of non-spike and spike-specific CD4^+^ and CD8^+^ T cell responses. PBMCs obtained before and 1-2 weeks after the third dose of CoronaVac were stimulated with spike peptide pools from WT, Delta, and Omicron as well as a non-spike peptide pool from WT SARS-CoV-2 for 20 h. **(A–C)** TNF-α and IFN-γ-producing CD4^+^ and CD8^+^ T cells in response to non-spike and spike peptides were detected by intracellular cytokine staining. **(A)** Data showed percentages of TNF-α and IFN-γ-producing CD4^+^ T cells against non-spike and spike peptides. **(B)** Data showed percentages of TNF-α and IFN-γ-producing CD8^+^ T cells against non-spike and spike peptides. **(C)** Cytokine-secreting CD4^+^ and CD8^+^ T cells against non-spike and spike peptides after the booster dose were calculated by aggregating amounts of TNF-α and IFN-γ-producing CD4^+^/CD8^+^ T cells, including TNF-α^+^IFN-γ^-^, TNF-α^+^IFN-γ^+^, and TNF-α^-^FN-γ^+^. Correlation analysis curve was plotted by the percentage of cytokine-secreting non-spike-specific CD4^+^ and CD8^+^ cells to their corresponding percentage of spike-specific CD4^+^ and CD8^+^ cells from WT, Delta, and Omicron. r, correlation coefficient. **(D)** Data showed percentages of CD107a^+^CD8^+^ T cells against non-spike and spike peptides determined by flow cytometry. Each point represents raw data for a single participant. Percentages show the percentage of T cells after subtracting the background value in a DMSO stimulation. Comparisons were performed *via* Wilcoxon matched-pairs signed rank test between two groups or Friedman test with Dunn’s multiple comparisons test among multiple groups. Correlation analysis was performed *via* nonparametric spearman correlation. n = 16 - 18. *p < 0.05, **p < 0.01, ***p < 0.001. NS, not significant.

Induction of cytokine-secreting non-spike and spike-specific CD4^+^ and CD8^+^ T cells was analyzed by intracellular cytokine staining assay (**Supplemental Figure 1**). The mean percentage of TNF-α-producing non-spike-specific CD4^+^ T cells was increased from 0.119% to 0.311% 1-2 weeks after the booster dose, while the proportion of IFN-γ-producing non-spike-specific CD4^+^ T cells was elevated from 0.019% to 0.047%. The booster dose also induced spike-specific TNF-α^+^CD4^+^ T cell responses of 0.289%, 0.332%, and 0.338% against WT, Delta, and Omicron, respectively, as well as spike-specific IFN-γ^+^CD4^+^ T cell responses of 0.038%, 0.049%, 0.051%, respectively (**Figure 2A**). On the other hand, the percentage of TNF-α-producing non-spike-specific CD8^+^ T cells was increased from 0.033% to 0.240%, while IFN-γ-producing non-spike-specific CD8^+^ T cells was raised from 0.016% to 0.112%. Spike-specific TNF-α^+^CD8^+^ T cell responses induced by the booster dose had a mean of 0.134%, 0.243%, and 0.328% against WT, Delta, and Omicron, respectively, while spike-specific IFN-γ^+^CD8^+^ T cell responses had a mean of 0.091%, 0.106%, and 0.144%, respectively (**Figure 2B**). Overall, no significant difference was observed in cytokine-secreting spike-specific CD4^+^ and CD8^+^ T cells among WT, Delta, and Omicron after booster dose. The data variation among the three groups may be attributed to baseline levels before vaccination and small sample size. The responses at baseline may represent the durable T cell immunity from prior 2 doses of homogenous vaccination and/or prior seasonal coronavirus infection. It is worth noting that the frequencies of cytokine-secreting non-spike-specific CD4^+^ and CD8^+^ T cells (TNF-α^+^IFN-γ^-^, TNF-α^-^IFN-γ^+^, and TNF-α^+^IFN-γ^+^) correlated well with those of spike-specific of wild-type, Delta, Omicron (**Figure 2C**). The association between spike and non-spike T cell response within each individual was less robust for the CD8 compared with that of CD4. Similar phenomenon was also reported by Zuo et al. in participants at 6 months following SARS-CoV-2 infection (12). In summary, the booster dose induced TNF-α and IFN-γ production in CD4^+^ and CD8^+^ T cells in response to both non-spike and spike antigens.

In addition, the frequency of non-spike CD107a^+^CD8^+^ T cells, reflective of cytotoxic function, was elevated from 0.249% to 0.381% after the booster dose (**Figure 2D**). In the meantime, spike-specific CD107a^+^CD8^+^ T cell response stimulated by the booster dose had a mean of 0.450%, 0.338%, and 0.528% against WT, Delta, and Omicron, respectively. Again, no difference was shown in CD107a^+^CD8^+^ T cells among WT, Delta, and Omicron. This finding indicates that the booster dose promoted the cytotoxic function of CD8^+^ T cells in response to both non-spike and spike antigens.

### Enhanced expression of activation-induced markers (AIM) by non-spike and spike-specific CD4^+^ and CD8^+^ T cells after the third dose

**Figure 3:**
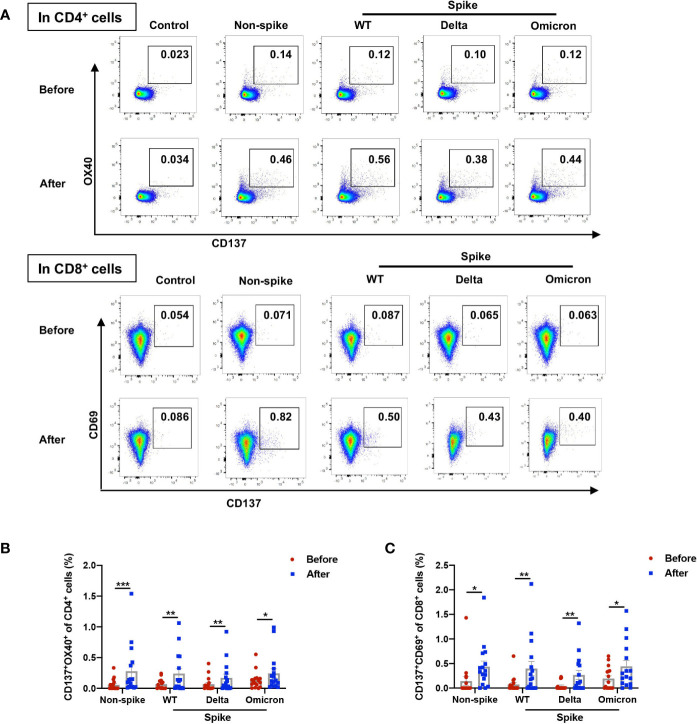
Non-spike and spike-specific AIM^+^CD4^+^ and AIM^+^CD8^+^ T cell responses after a third dose of CoronaVac. PBMCs obtained before and 1-2 weeks after the third dose of CoronaVac were stimulated with spike peptide pools from WT, Delta, and Omicron as well as a non-spike peptide pool from WT SARS-CoV-2 for 20 h. The expression of AIM on CD4^+^ (CD137^+^OX40^+^) and CD8^+^ (CD137^+^CD69^+^) T cells was measured by flow cytometry. **(A)** Representative flow cytometry plots showed the frequency of AIM^+^CD4^+^ and AIM^+^CD8^+^ T cells. **(B)** Percentages of AIM^+^ (CD137^+^OX40^+^) cells among CD4^+^ T cells responding to non-spike and spike peptides were displayed. **(C)** Percentages of AIM^+^ (CD137^+^CD69^+^) cells among CD8^+^ T cells responding to non-spike and spike peptides were displayed. Each point represents raw data for a single participant. Percentages represent the percentage of T cells after subtracting the background value in a DMSO stimulation. Comparisons were performed *via* Wilcoxon matched-pairs signed rank test between two groups or Friedman test with Dunn’s multiple comparisons test among multiple groups. n = 16. *p < 0.05, **p < 0.01, ***p < 0.001.

To further capture the characteristics of viral-specific T cell response, AIM assay was adopted to study T cell response. The AIM assay identifies early-responding T cells and is independent of cytokine production (8). It was reported that the percentage of AIM^+^CD4^+^ T cells did not have a strong correlation with the frequency of cytokine-producing CD4^+^ T cells measured by intracellular cytokine staining or ELISpot (13). The expression of AIM on CD4^+^ (CD137^+^OX40^+^) and CD8^+^ (CD137^+^CD69^+^) cells was measured by flow cytometry (**Figure 3A** and **Supplemental Figure 2**). The booster dose increased the proportion of AIM^+^CD4^+^ cells from 0.057% to 0.280% in response to the non-spike peptide pool. When stimulated with spike peptide pools from WT, Delta, and Omicron, the percentages of AIM^+^CD4^+^ cells 1-2 weeks after the booster dose were elevated to 0.242%, 0.170%, and 0.244%, respectively, indicating substantial cross-reactivity of CD4^+^ T cell responses between WT and variants (**Figure 3B**). The frequency of AIM^+^CD8^+^ cells was augmented from 0.144% to 0.435% upon the stimulation with the non-spike peptide pool. The booster dose increased the frequency of AIM^+^CD8^+^ cells to 0.398%, 0.267%, and 0.442% against WT, Delta, and Omicron, respectively, which also indicates substantial cross-reactivity of CD8^+^ T cell responses between WT and variants (**Figure 3C**).

### Subset distribution of non-spike and spike-specific CD4^+^ and CD8^+^ T cells after a third dose of CoronaVac

**Figure 4:**
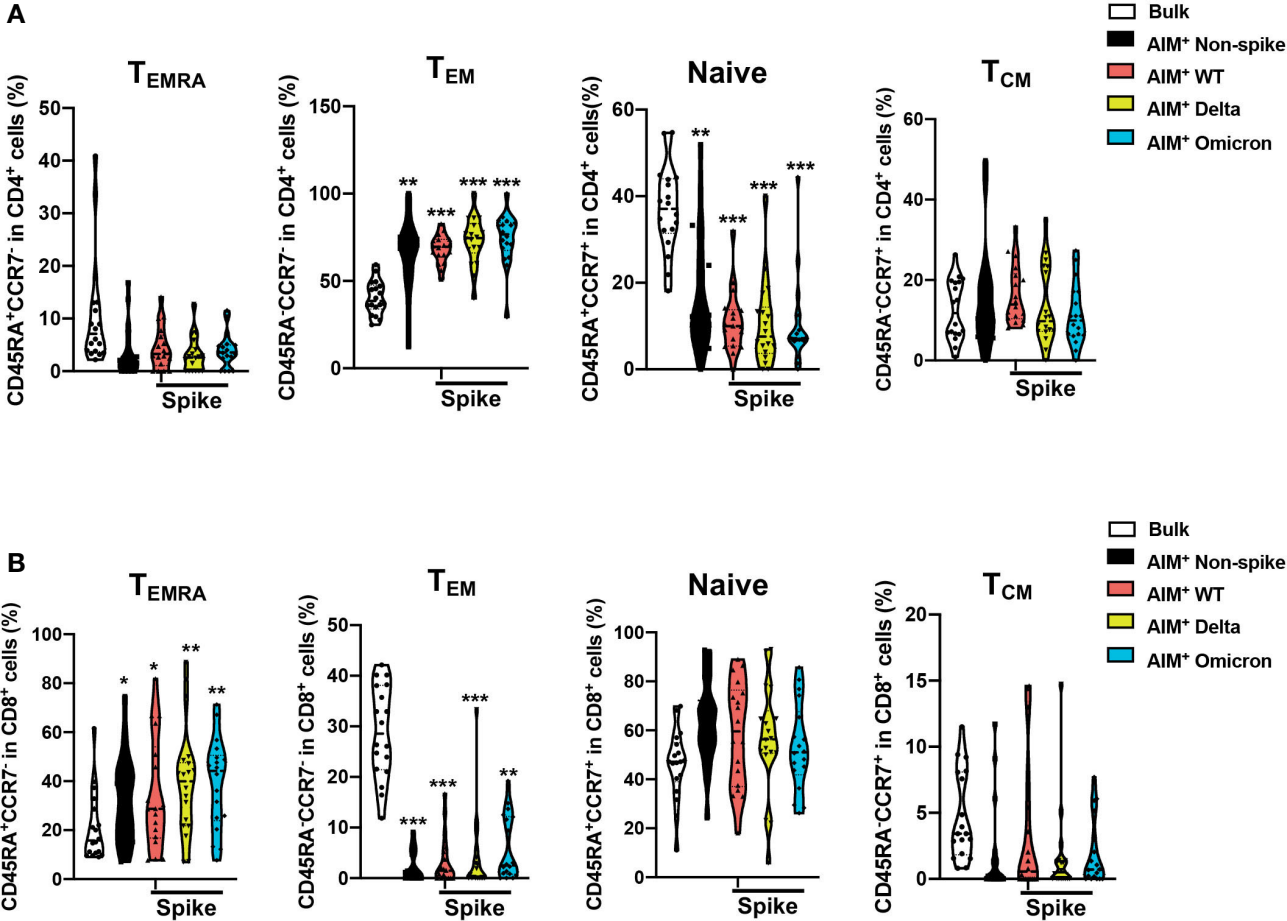
Distribution of memory T cell subsets among SARS-CoV-2–specific CD4^+^ and CD8^+^ T cells after a third dose of CoronaVac. The percentages of naive (CD45RA^+^CCR7^+^), central memory (T_CM_, CD45RA^-^CCR7^+^), effector memory (T_EM_, CD45RA^-^CCR7^-^), and terminally differentiated effector memory (T_EMRA,_ CD45RA^+^CCR7^-^) in the bulk and SARS-CoV-2 AIM^+^ populations for CD4^+^ **(A)** and CD8^+^ **(B)** T cells were determined by flow cytometry. The percentages of T memory subsets in the AIM^+^ populations were compared with those of bulk CD4^+^ and CD8^+^ cells. Comparisons were performed *via* RM one-way ANOVA with Holm-Šídák’s multiple comparisons test for parametric data or Friedman test with Dunn’s multiple comparisons test for nonparametric data. n = 18. *p < 0.05, **p < 0.01, ***p < 0.001.

To assess the differentiation status of AIM^+^ T cells after the booster dose, the percentages of naive (CD45RA^+^CCR7^+^), central memory (T_CM_, CD45RA^-^CCR7^+^), effector memory (T_EM_, CD45RA^-^CCR7^-^), and terminally differentiated effector memory (T_EMRA_, CD45RA^+^CCR7^-^) in the bulk and SARS-CoV-2 AIM^+^ populations of CD4^+^ and CD8^+^ T cells were examined *via* flow cytometry (**Supplemental Figure 2** for gating strategy). CD4^+^ and CD8^+^ T cells were enriched preferentially for the T_EM_ and naïve subsets in the bulk (**Figures 4A, B**). Both non-spike and spike-specific AIM^+^CD4^+^ T cells exhibited a significant increase in the T_EM_ population and a significant decrease in the naïve population compared with bulk CD4^+^ cells (**Figure 4A**). Compared with the bulk CD8^+^ cells, non-spike and spike-specific AIM^+^CD8^+^ had reduced expression of T_EM_ and elevated expression of T_EM_ re-expressing CD45RA (T_EMRA_), the most differentiated subset of human CD8^+^ T cells (**Figure 4B**). In contrast to CD8^+^ T_EM_, CD8^+^ T_EMRA_ has elevated expression of perforin and granzyme B and is capable of killing specific target cells without prior activation (14). These results suggest that non-spike and spike-specific AIM^+^CD4^+^ and AIM^+^CD8^+^ T cells develop phenotypes of activation and functional capacity after the booster dose.

## Discussion

COVID-19 vaccination induces neutralization antibodies, effector T cell responses, and memory T cell responses for viral antigens. In the present study, we conducted an examination of T cell responses after a homogenous booster (third) dose of an inactivated vaccine, CoronaVac. Our results showed that the booster dose induced cytokine production in CD4^+^ and CD8^+^ T cells as well as cytotoxicity of CD8^+^ T cells after stimulation with non-spike or spike antigens. In addition, non-spike-specific cytokine responses in CD4^+^ and CD8^+^ T cells correlated well with the counterparts for spikes from WT, Delta, and Omicron. AIM^+^CD4^+^ and AIM^+^CD8^+^ T cell responses were elevated for non-spike and broadly conserved toward WT, Delta, and Omicron. In the meantime, booster vaccination expanded CD4^+^ T_EM_ as well as CD8^+^ T_EMRA_ subsets of non-spike and spike-specific T cells.

Several studies have been conducted to study spike-specific T cell response after booster dose of inactivated vaccine. Li et al. reported that the frequency of AIM^+^ (CD69^+^ CD137^+^) spike‐specific total T cells was significantly increased for both WT and Omicron after the third dose of CoronaVac, but the enhancement was diminished in the Omicron. Memory T cell subsets were examined in AIM^+^ total T cells (CD69^+^CD137^+^CD45RO^+^CD45RA^‐^), while no difference was observed in intergroup comparisons (15). Schultz et al. documented that a booster dose of CoronaVac elevated the percentage of AIM^+^ (OX40^+^ CD137^+^) CD4^+^ T cells against the Delta and Omicron, comparable to the WT strain (16). Chen et al. found that AIM^+^CD4^+^ and AIM^+^CD8^+^ T cell recognition against the Delta and Omicron was slightly reduced but largely maintained, when compared with the reference ancestral strain, after a booster dose of CoronaVac (17). Xiao et al. showed that spike-specific CD8^+^ T cells for recognizing Delta and Omicron variants were lower than that of ancestral strain with booster dose of inactivated vaccine KCONVAC (18). The present study filled a knowledge gap by examining non-spike T cell response and memory cell subsets.

The present study used a peptide pool from WT non-spike proteins to stimulate CD4^+^ and CD8^+^ T cell responses. Tarke et al. has demonstrated a broad pattern of immunodominance for SARS-COV-2. Eight to nine proteins including non-structural protein (nsp)3, nsp4, nsp12, open reading frame 3a (ORF3a), S, M, and N are required to induce 80% of CD4 and CD8 T cell responses (19). They also pointed out that S, N, and M may have immunodominance due to high abundance. The non-spike peptide pool in the present study was derived from 7 proteins (N, M, ORF1, nsp3, ORF-3a, ORF-7a, and ORF8) which has a significant overlap with the reported immunodominant proteins abovementioned. Although the non-spike peptide pool did not pinpoint the individual antigen of immunodominance, it serves to compare non-spike as a whole with spike in the present study. Similar non-spike peptide pool approach has also been reported by other labs (20).

Using intracellular cytokine staining assay, Rosa Duque et al. reported that two doses of BNT162b2 and CoronaVac generated similar spike-specific T cell responses to WT SARS-CoV-2, while non-spike T cell response was only detected in CoronaVac due to lack of non-spike antigens in BNT162b2 (21). There are several differences between the aforementioned study and the present study. We examined non-spike and spike-specific memory T cell responses after the booster of CoronaVac. The methodologies for our study were more extensive and included ELISpot, intracellular cytokine staining, and AIM assay. In addition, we examined the T cell response not only against WT but also Delta and Omicron.

CD8^+^ T cells directly kill virus-infected cells and generate proinflammatory cytokines and chemokines, which attract additional immune cells to sites of infection. CD4^+^ T cells facilitate the expansion and function of both B cells and CD8^+^ T cells. They also possess antiviral properties similar to CD8^+^ T cells by producing proinflammatory cytokines and killing *via* direct cytolytic actions (22). Naranbhai et al. showed that T cell immunity to the Omicron variant was preserved in the majority of infected and vaccinated individuals (11). Redd et al. found that there was minimal crossover between mutations in the Omicron variant and viral epitopes recognized by CD8^+^ T in COVID-19 convalescent patients indicating that cell responses should recognize the Omicron variant (23). In patients with multiple sclerosis receiving ocrelizumab, T cell responses were negligibly affected by the Omicron variant and may prevent the occurrence of severe COVID-19 (24). Gao et al. found that spike-specific CD4^+^ and CD8^+^ T cells responses induced by BNT162b2 vaccination or prior SARS-CoV2 infection were largely intact against Omicron B.1.1.529 (25). Keeton et al. discovered that the magnitude of spike-specific CD4^+^ and CD8^+^ T cell responses to Omicron B.1.1.529 was mostly sustained and similar to those of Beta and Delta variants in COVID-19 convalescent patients and participants with vaccination of Ad26.CoV2.S or BNT162b2 (26). In participants with Ad26.COV2.S or BNT162b2 vaccination, Liu et al. demonstrated that spike-specific CD8^+^ and CD4^+^ T cell responses were long-lasting and extensively cross-reactive for both the Delta and Omicron B.1.1.529 (27). In health care workers with vaccination of ChAdOx-1 S, Ad26.COV2.S, mRNA-1273, or BNT162b2, GeurtsvanKessel et al. revealed that there was no significant difference in spike-specific CD4^+^ and CD8^+^ T cell responses between WT and variants including Delta, Beta, and Omicron B.1.1.529 (28). In volunteers with diverse vaccination and SARS-CoV-2 infection backgrounds, De Marco et al. showed that approximately 87% of cellular immunity was maintained for S protein of the Omicron BA.1 (29). The present study also showed that the booster dose elicited cross-reactivity of functional CD4^+^ and CD8^+^ responses between WT and variants.

SARS-CoV-2 infection induces a wide spectrum of antigen-specific T cells. Fazolo et al. showed that pediatric COVID-19 patients had higher TNF^+^CD8^+^ T cell response for the M and N antigens compared with that of spike (30). They also found that N-specific TNF^+^CD8^+^ T cell response in pediatric COVID-19 patients was sustainable and had T_EM_ and T_EMRA_ phenotypes (31). Kundu et al. demonstrated that SARS-CoV-2 infection was lower in individuals with non-spike memory T cells reactive to ORF1 and N proteins (32). In convalescent COVID-19 patients, Ferretti et al. showed that the majority of epitopes recognized by CD8 T cells were localized in ORF1ab and the N protein (33). In convalescent individual following COVID-19, there was a broad and strong memory T cell response against spike, non-spike structural proteins, and non-structural proteins (8). The present study demonstrated that booster dose of CoronaVac elicited both non-spike and spike-specific CD4^+^ and CD8^+^ T cell responses. In addition, the induced spike-specific T cell response was cross-reactive toward WT, Delta, and Omicron. Therefore, second-generation mRNA vaccines targeting non-spike proteins might provide more protection against the Omicron variants.

The phenotypic features of SARS-CoV-2-specific CD4^+^ and CD8^+^ T cells after administration of inactivated vaccine are poorly understood. The present study demonstrated that the proportion of T_EM_ for both non-spike and spike-specific AIM^+^CD4^+^ T cells was elevated after the third dose of CoronaVac. In the meantime, the population of T_EMRA_ for AIM^+^CD8^+^ T cells was increased, regardless of the variant peptide analyzed. This finding is similar to previous reports examining T cell phenotypes after SARS-CoV-2 infection and mRNA vaccination. In studying memory response to SARS-CoV-2 infection, Dan et al. found that the majority of SARS-CoV-2-specific AIM^+^CD4^+^ T cells was T_CM_ and T_EM_ with small percentage of T_EMRA_. However, the majority of SARS-CoV-2-specific AIM^+^CD8^+^ T cells was T_EMRA_ with small populations of T_CM_ and T_EM_ (34). In a study of infected and mRNA-vaccinated individuals, spike-specific AIM^+^ T cells, irrespective of the variant peptide analyzed, showed enrichment for T_CM_ and T_EM_ subsets for CD4^+^ T cells and T_EMRA_ subset for CD8^+^ T cells (35). Rodda et al. reported that CD4^+^ T_EM_ cells from individuals recovered from COVID-19 had the ability to proliferate and re-seed the memory pool upon antigen re-exposure, providing protection against re-infection (36). In 2009 H1N1 pandemic, CD8^+^ T_EMRA_ cells were inversely correlated with symptom score and had cytotoxic potential (37). Our findings indicate that the booster dose of CoronaVac produces non-spike and spike-specific memory T cell responses that recognize variants and provide protection against infection.

Our study has several limitations. First, cellular immune response to non-spike peptide pool for Omicron was not examined in the study due to commercial unavailability. Ahmed et al. reported that 98% of 745 non-spike CD8^+^ T cell epitopes and 95% of 373 non-spike CD4^+^ T cell epitopes were unaffected by mutations in Omicron (B.1.1.529) (38). Therefore, we postulate that booster vaccination of inactivated COVID-19 vaccines would elicit similar non-spike-specific CD4^+^ and CD8^+^ T cell responses between wild-type and Omicron. Second, the sample size was small with 18 volunteers. A large sample size would provide higher statistical power in deciphering non-spike and spike-specific T cell responses. Third, the time point to assess immune response was conducted at 1-2 weeks after booster dose. Wang et al. demonstrated that non-spike and spike T cell responses as assayed by ELISPOT were readily detectable 6 months after the third dose of inactivated vaccine BBIBP-CorV (Covilo). A homologous booster 6 months after the third dose failed to further enhance T cell responses (39). Melo-González et al. reported that AIM^+^CD4^+^ T cell response remained stable after the third dose of CoronaVac, but showed a decline 4-6 months later (40). The durability of cellular immune response after booster dose of CoronaVac warrants further investigation. Furthermore, this study was conducted in healthcare workers which skewed toward a healthy and young population.

## Conclusions

The third dose of inactivated COVID-19 vaccine enhances cellular immune response against SARS-CoV-2. The booster dose elicits cytokine and AIM responses in CD4^+^ and CD8^+^ T cells towards non-spike and spike peptide antigens from SARS-CoV-2 of WT, Delta, and Omicron. Future COVID-19 vaccine formulations containing non-spike components may offer better coverage against variants.

## Data availability statement

The raw data supporting the conclusions of this article will be made available by the authors, without undue reservation.

## Ethics statement

The studies involving human participants were reviewed and approved by The Children’s Hospital of Zhejiang University School of Medicine. The patients/participants provided their written informed consent to participate in this study.

## Author contributions

RH: Conceptualization (equal); Data curation (lead); Formal analysis (equal); Funding acquisition (equal); Methodology (equal); Writing-original draft (lead); and Writing-review and editing (equal). LY: Conceptualization (equal); Data curation (lead); Formal analysis (equal); Methodology (equal); Writing-original draft (lead); and Writing-review and editing (equal). JW, JX, YZ, HM, RYZ, RXZ, and ZL: Data curation (equal); Formal analysis (equal); Methodology (equal); Validation (equal); and Writing-review and editing (equal). QS and JGX: Conceptualization (equal); Formal analysis (equal); Funding acquisition (equal); Supervision (equal); Writing-original draft (equal); and Writing-review and editing (equal). All authors contributed to the article and approved the submitted version.

## Supplementary material

Supplementary Figure 1 Gating strategy for determining cytokine-producing cells among CD4^+^ and CD8^+^ T cells as well as cytotoxic CD107^+^CD8^+^ T cells.

Supplementary Figure 2 Gating strategy for determining AIM^+^CD4^+^ (CD137^+^OX40^+^) and AIM^+^CD8^+^ (CD137^+^CD69^+^) memory T cells as well as their subpopulations.
