# Supplementary material for: Non-spike and spike-specific memory T cell responses after the third dose of inactivated COVID-19 vaccine
Source: Front Immunol. 2023 Apr 11;14:1139620. doi: 10.3389/fimmu.2023.1139620 (PMC10126277; doi:10.3389/fimmu.2023.1139620)
Supplement: Supplementary Figure 1 — Gating strategy for determining cytokine-producing cells among CD4+ and CD8+ T cells as well as cytotoxic CD107+CD8+ T cells. [file Image_1.pdf]

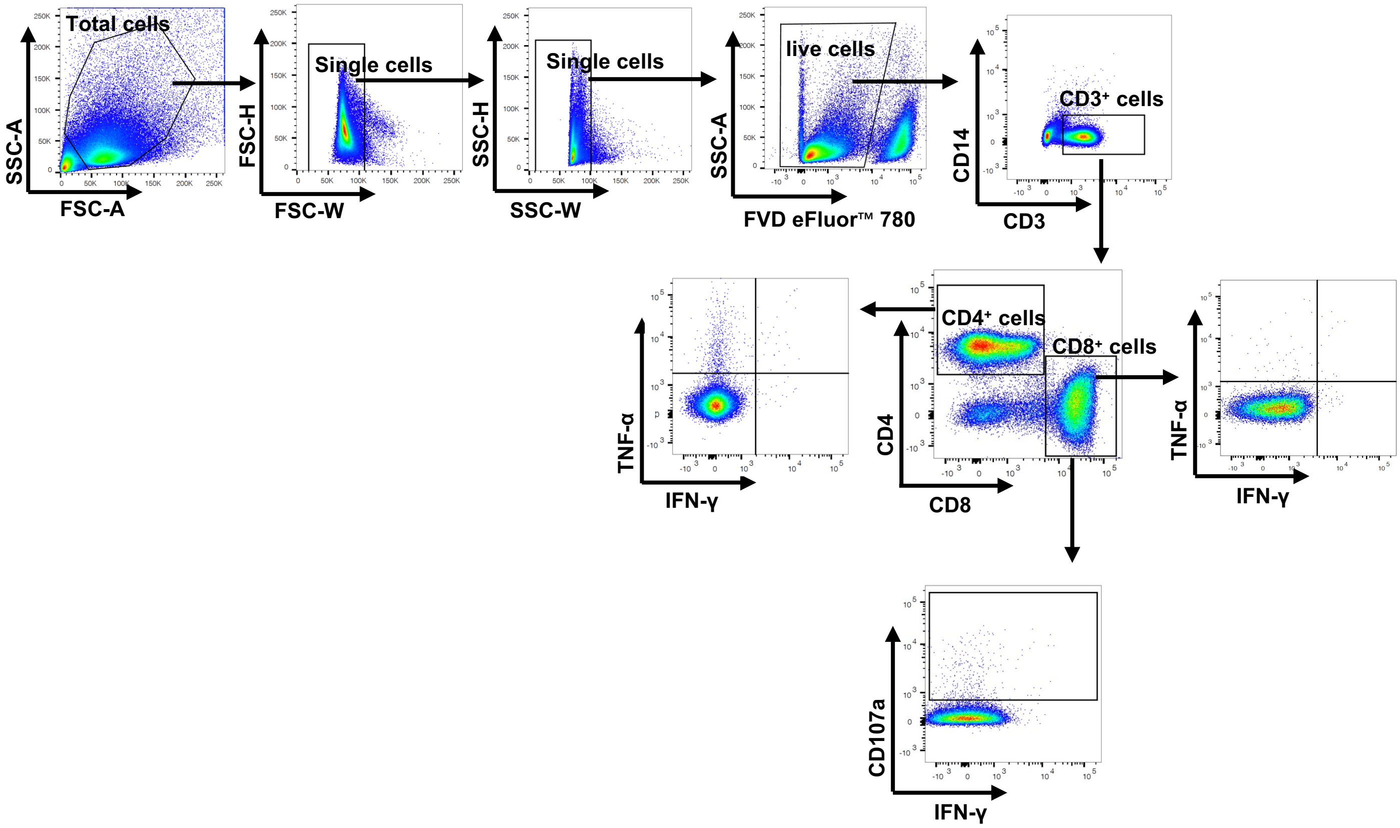

Supplemental Figure 1. Gating strategy for determining cytokine-producing cells among CD4$^+$ and CD8$^+$ T cells as well as cytotoxic CD107$^+$CD8$^+$ T cells.
