# Supplementary material for: Non-spike and spike-specific memory T cell responses after the third dose of inactivated COVID-19 vaccine
Source: Front Immunol. 2023 Apr 11;14:1139620. doi: 10.3389/fimmu.2023.1139620 (PMC10126277; doi:10.3389/fimmu.2023.1139620)
Supplement: Supplementary Figure 2 — Gating strategy for determining AIM+CD4+ (CD137+OX40+) and AIM+CD8+ (CD137+CD69+) memory T cells as well as their subpopulations. [file Image_2.pdf]

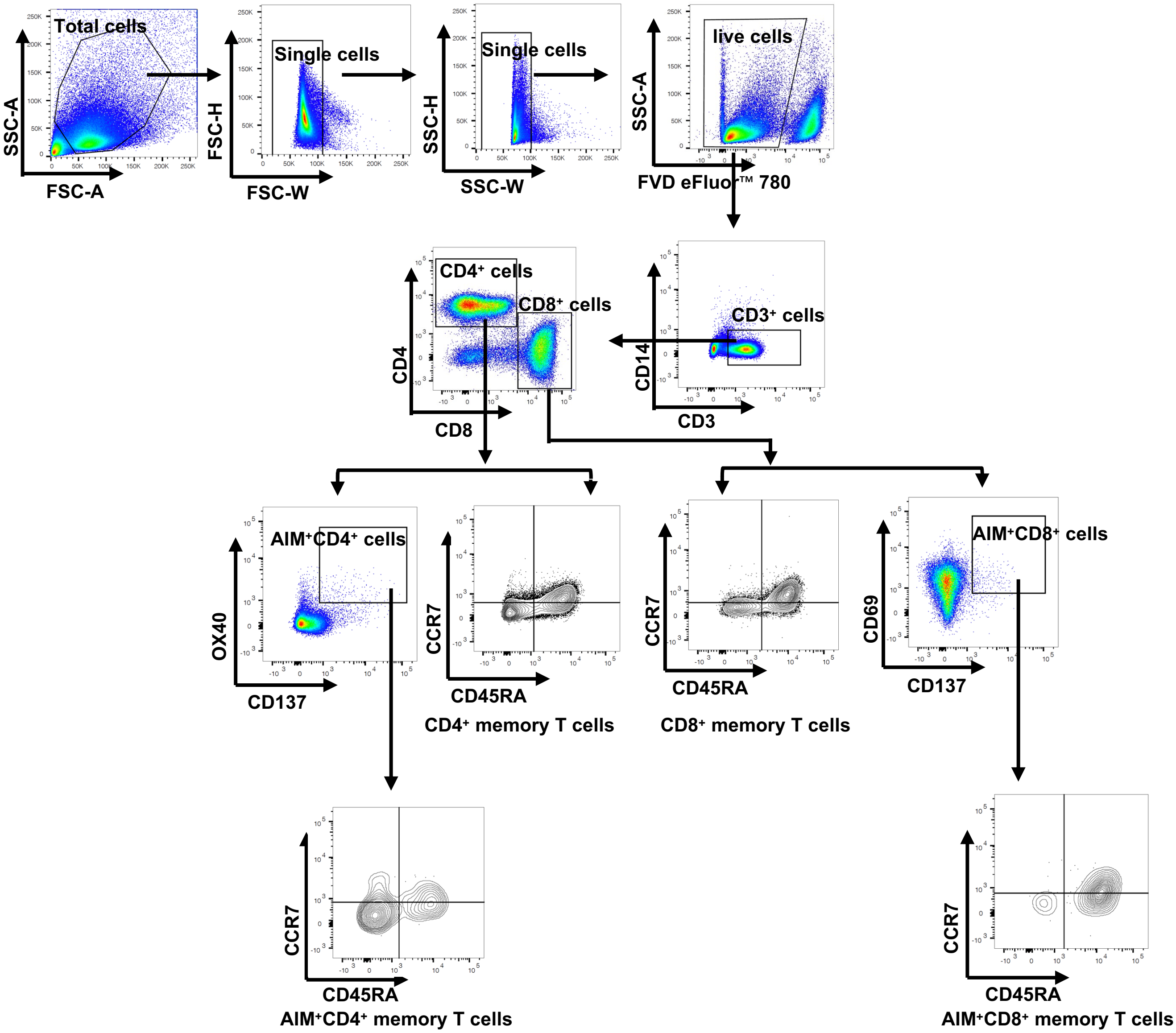

Supplemental Figure 2. Gating strategy for determining AIM^{+}CD4^{+} (CD137^{+}OX40^{+}) and AIM^{+}CD8^{+} (CD137^{+}CD69^{+}) memory T cells as well as their subpopulations.

Supplemental Figure 2
